# Generation of Kazakhstan's unified national testing variants using AI: a platform for automatic task creation with expert control

**DOI:** 10.3389/fdata.2026.1772101

**Published:** 2026-04-17

**Authors:** Bolatbek Abdrasilov, Talgat Niyazov, Lyazzat Shinetova, Shugyla Altybayeva, Kenzhekul Turalbayeva, David Orlov

**Affiliations:** National Testing Center, Kazakhstan, Astana, Kazakhstan

**Keywords:** AI, AIG, automated item generation, English, Kazakh, KazBERT, KenesBERT, large language models

## Abstract

This study examines the use of artificial intelligence for Automatic Item Generation (AIG) in the context of Kazakhstan's Unified National Testing (UNT) and presents a human-in-the-loop platform for scalable, expert-controlled test development. The objective is to evaluate whether large language models (LLMs) can reliably generate high-quality, isomorphic mathematics test items in the Kazakh language while preserving psychometric and pedagogical requirements. A hybrid AI system combining a local and a cloud-based LLM was implemented to perform semantic deconstruction of prototype items and constrained isomorphic generation of new variants. The pipeline included structured prompt engineering, parallel generation, and automated symbolic validation using Python and SymPy, followed by double-blind expert review. A stratified sample of 120 UNT mathematics items served as prototypes, from which 200 AI-generated clones were produced and validated. Six qualified subject-matter experts conducted independent evaluations using standardized criteria. Inter-rater reliability reached a substantial level (Cohen's κ = 0.78). Results show that 97.5% of generated items were recommended for use after review, with 50.5% accepted without revision and 47.0% accepted after corrections. The most frequent revision needs involved difficulty calibration, wording clarity, and factual or curricular alignment. Expert interviews confirmed that AI generation significantly reduces development time but remains limited in higher-order cognitive item design and pedagogically grounded distractor construction, especially in a low-resource, morphologically complex language environment. The findings support a hybrid augmentation model in which AI accelerates large-scale item production while experts ensure linguistic, cultural, and psychometric validity. The proposed framework demonstrates practical potential for multilingual, high-stakes assessment systems and provides implementation guidelines for responsible AI integration in test development.

## Introduction

Digitalization of education is transforming traditional approaches to assessment. Artificial intelligence (AI) technologies reinforce this transformation by enabling the creation of systems that automate various stages of assessment. This responds to the growing need for more adaptive, scalable, and secure forms of knowledge measurement (OECD, 2023a,b).

A promising direction of the digital transformation of assessment is automated item generation (AIG). Classical studies show that AIG makes it possible to create a large number of high-quality items based on a single cognitive model, reducing expert labor costs (Gierl and Haladyna, 2012; Gierl et al., 2016). Contemporary research demonstrates that the use of large language models (LLMs) significantly expands the capabilities of AIG, enabling the generation of content-appropriate and psychometrically valid items (Circi et al., 2023; Sangiovanni et al., 2023; Tong and Krittayaphongphun, 2024). International organizations, including the OECD, emphasize the strategic importance of digital item banks and the adoption of innovative technologies to ensure the scalability and quality of assessment (OECD, 2023a).

International experience confirms the active implementation of AI technologies in assessment systems. One of the most advanced examples is the Educational Testing Service (ETS, USA), where AI is used in several key areas: AIG, scoring of written responses using the e-Rater system (Burstein et al., 2003), automated assessment of spoken language using the SpeechRater service, as well as the planning and modeling of adaptive testing. At the same time, ETS adheres to a “human-in-the-loop” model: any decisions proposed by algorithms are subject to mandatory expert review and may be adjusted by specialists. This approach ensures a balance between technological advancement and the psychometric reliability of results (Deane and Higgins, 2019; ETS Global, 2024).

AQA and related examination bodies (OxfordAQA, 2024; JCQ, 2024, 2025) treat any content generated by AI—including text, images, and graphics—as the work of a third party. If a student includes such content in an assignment, they are required to explicitly acknowledge that it is AI-generated. At present, AQA considers the automated generation of examination items and automated scoring to be acceptable only as supporting tools and requires human oversight. For high-stakes examinations, such as GCSE and A-level, the large-scale use of unsupervised AI is considered unacceptable.

In China, numerous research projects have explored the use of AI for analyzing, classifying, generating, and estimating the difficulty of test items. Several studies report successful applications of neural network models using datasets derived from Gaokao test items, the national university entrance examination (Yang et al., 2018; Tang et al., 2021; Zhang et al., 2022; Ding et al., 2023; Qi et al., 2020). At the same time, there is no publicly available official evidence of state-level integration of AI into the development of Gaokao examination materials; AI is used primarily for research and technological optimization purposes.

The development of AIG technologies reinforces the global trend toward the introduction of adaptive testing, as such systems require large and regularly updated item banks (Gierl and Lai, 2013a,b). Despite substantial progress, unresolved challenges remain, most notably the development of higher-order cognitive items, where AIG continues to demonstrate limited capability (Gierl and Bulut, 2021; Circi et al., 2023). Critical reviews of the literature also identify an important gap: the vast majority of AIG research has been conducted in English-language contexts, where well-developed item repositories and testing standards provide more favorable conditions for automation (Circi et al., 2023; Bulut and Gierl, 2022).

### The assessment context in Kazakhstan

The issue of adapting AI technologies is particularly relevant in multilingual and culturally specific education systems, such as that of Kazakhstan. In the Republic of Kazakhstan, the education system is regulated by the Law “On Education,” (Republic of Kazakhstan, 2007) which establishes an assessment framework emphasizing the integration of national standards, modern technologies, and requirements for objectivity and procedural transparency. This legislation defines a trilingual education model, comprising Kazakh as the state language, Russian as the language of interethnic communication, and one foreign language. At the same time, the right to receive education in one's native language is guaranteed; in practice, this results in the operation of schools offering instruction in Kazakh, Russian, Uzbek, Uyghur, Tajik, and other languages.

The key assessment instrument in Kazakhstan is the Unified National Testing (UNT), administered by the National Testing Center (NTC) (OECD, 2020; Abdrasilov et al., 2024).

The UNT is a computer-based test consisting of 120 items across five subjects, with a total testing time of 240 min. In recent years, the system has undergone substantial modernization, including a full transition to computer-based testing (CBT), the introduction of AI-based proctoring, and an increase in the number of permitted test attempts (Abdrasilov et al., 2024; Altybaeva et al., 2025; Altybaeva and Skabaeva, 2025; Didarbekova et al., 2025). In 2025, the test was taken 728,983 times (National Testing Center, 2025).

However, a significant technological gap exists between test delivery and test development. While the administration of the UNT is fully digitized, the development of test items remains a labor-intensive manual process. This inefficiency becomes particularly critical given the need for frequent renewal of the item bank to support five testing periods per year (Abdrasilov et al., 2025; Altybaeva et al., 2025; Didarbekova et al., 2025). This gap creates a practical demand for the adoption of technologies such as AIG.

The UNT is administered in Kazakh, Russian, and English, giving rise to a complex methodological challenge (Abdrasilov et al., 2024; Didarbekova et al., 2025). It is necessary to ensure not only linguistic translation, but also semantic, cultural, didactic, and psychometric equivalence across test versions. Consequently, the Kazakhstani context constitutes a unique research setting for piloting AIG technologies within a complex multilingual and multicultural educational environment.

### Analysis of existing research

The study Impact of Language of Instruction on Progress in Kazakhstan (Nurseitova et al., 2017) shows that the language of instruction has a significant effect on UNT outcomes, highlighting the importance of accounting for language-related factors in Kazakhstan's multilingual education system. At the same time, limitations of existing research indicate that ensuring fairness in assessment within a multilingual context requires consideration not only of language, but also of a broader sociocultural and didactic context. Thus, the task of adapting AI technologies extends beyond simple linguistic translation and requires attention to deeper sociocultural and didactic factors.

The integration of LLMs into the generation and scoring of test items is associated with substantial risks. Yan et al. (2024) identify key challenges, including insufficient trust in automatically generated content, latent algorithmic biases, and the blurring of responsibility among stakeholders. For high-stakes examinations such as the UNT, these challenges are particularly critical.

The development of AI technologies for the Kazakh language faces challenges common to low-resource languages. Kazakh has a rich morphological structure, is agglutinative, and exhibits high variability in word forms, which substantially complicates the development of language models. In addition, the volume of annotated corpora remains limited, directly affecting the quality of text generation and processing. These challenges are global in nature and are well-documented in studies of other Turkic and indigenous languages, including Kyrgyz and Quechua. For example, research on the development of KyBERT models for the Kyrgyz language (Turdaly KyBERT Team, 2022), as well as studies conducted within the AmericasNLP shared tasks on processing indigenous languages of South America (Oncevay et al., 2021–2023), demonstrate similar issues, including data scarcity, morphological complexity, and difficulties in developing general-purpose NLP solutions for educational applications.

These challenges have prompted specialized initiatives aimed at developing Kazakh-language models. The development of KazBERT (Sakenov et al., 2021) and KenesBERT (Akhmetov et al., 2023) demonstrates that adapting BERT-based architectures can improve the quality of text understanding and generation in Kazakh, as well as enable the creation of tools sensitive to Kazakhstan's linguistic and cultural context. These models provide a foundation for the subsequent integration of AI into assessment systems: a high-quality language foundation is a necessary condition for developing test item generators, feedback-oriented chat models, automated scoring systems, and tools for diagnosing language competencies.

Progress in Kazakh speech recognition and synthesis also provides a foundation for the adoption of multimodal approaches. A review of contemporary Kazakh ASR/TTS technologies (Tussupova et al., 2023) shows that combined models can substantially enhance the effectiveness of assessing speaking and speech perception, which is particularly important for language proficiency tests. Thus, adapting AI technologies to Kazakhstan's multilingual context goes far beyond simple linguistic translation and requires consideration of morphology, discourse norms, and cultural features that influence learning and assessment processes.

The NTC conducted the first pilot study to evaluate the performance of large language models (LLMs) on UNT test items. Kadyrov et al. (2025) assessed six contemporary models using 139 mathematics items. The models Gemini, DeepSeek, Qwen, and o1 achieved high accuracy levels (91.2%−100% in a zero-shot setting), whereas Claude and Llama demonstrated substantially lower performance (43.5%−76.5%). These results indicate the potential of AI for assessment-related applications, while also highlighting the importance of adapting models to the linguistic and subject-matter context of Kazakhstan.

The position of the NTC regarding the adoption of AI remains cautious and exploratory. Strategic plans envisage examining the potential of AI as a supporting tool for automating routine operations, while the full replacement of human experts is not under consideration. This cautious stance underscores the need for scientifically grounded research capable of assessing both the risks and the potential of AIG specifically in the context of Kazakhstan, which defines the objective of the present study.

## Materials and methods

### Research materials

The study utilized multiple-choice mathematics items from the 2024 UNT item bank in the Kazakh language. A representative corpus of ***N*** **=** **120 items** was constructed through stratified random sampling according to the official **UNT-2024 test specification**. The sampling ensured proportional coverage of content domains (algebra, geometry, introductory calculus) and cognitive complexity levels (knowledge/understanding, application, analysis/synthesis). These items served as **reference prototypes** for subsequent automated isomorphic cloning.

### Automated item generation (AIG) procedure

The core methodological approach was **controlled template-based isomorphic generation**. This method preserves the invariant logical-mathematical core of an item, defined by the assessed skill, solution structure, and response format, while algorithmically varying its surface features, including numerical parameters, contextual elements, and lexical formulation.

#### Implementation in the “AI TestGen NCT” system

The AIG pipeline was implemented in a custom hybrid system (“AI TestGen NCT”) with the following components:

1. **Generative Architecture:** A dual-LLM setup was employed for parallel generation and diversification:
  - **Local Model:** GPT Oss 120b (quantized, run locally for data privacy and semantic deconstruction).
  - **Cloud Model:** Google Gemini 2.5 Pro (accessed via OpenRouter API for state-of-the-art generation capabilities).
2. **Generation Pipeline:**
  (a) **Semantic Deconstruction:** Each reference prototype was analyzed by the local LLM to extract its semantic “core”: the specific skill identifier (Skill ID), the step-by-step solution logic, and the pedagogical rationale behind each distractor (typical student error).
  (b) **Prompt-Based Specification:** The extracted core was formatted into a structured, machine-readable **technical specification** (JSON-based prompt) defining constraints for isomorphism.
  (c) **Parallel Isomorphic Generation:** The specification was sent simultaneously to both LLMs to generate new item variants. The prompts embedded four strict **isomorphism criteria**: (1) **Conceptual Invariance**, (2) **Structural Similarity**, (3) **Equivalence of Difficulty**, (4) **Didactic Adequacy of Distractors**.
  (d) **Technical Parameters:** Generation temperature was set to **0.65** to balance creativity and consistency. Output was forced into a structured **LaTeX (exam class)** format in Kazakh.
3. **Automated Validation and Corpus Construction:** Generated items were programmatically validated using **Python 3.11** with the **SymPy (v1.12)** library for symbolic computation. The validation script:
  - Parsed the LaTeX output to extract numerical data and equations.
  - Computed the correct answer independently.
  - Compared the computed result with the LLM-provided answer key.

Items failing validation were either regenerated or manually corrected. This process yielded the final experimental corpus of N = 200 valid, cloned items for expert evaluation.

**Structured prompt design**. To operationalize the isomorphic generation framework, two specialized text-based instruction templates (prompts) were developed. These prompts served as formal interfaces between the UNT assessment specifications and the generative LLMs, translating pedagogical requirements into executable model instructions.

1. **Feature Extraction and Semantic Deconstruction Prompt**. This prompt was designed to guide the local LLM (GPT Oss 120b) in performing an analytical decomposition of the source item. Its primary function was to convert an item's textual representation into a structured, machine-readable specification. The prompt instructed the model to perform the following analytical tasks:
  - Identify and code the item's primary assessed skill according to the official UNT content domain and topic taxonomy.
  - Determine the specific learning objective code from the State Educational Standard that the item targets.
  - Reconstruct the logical sequence of steps constituting the correct solution path.
  - Analyze each multiple-choice option to classify it as the correct answer or to specify the exact type of student misconception or reasoning error that a distractor is intended to capture.
  - Output this structured analysis in a predefined JSON format, creating a formal “item specification” that encapsulates the invariant core.
2. **Constrained Isomorphic Generation Prompt**. This prompt provided a rule-based generation template for the cloud-based LLM (Gemini 2.5 Pro). It received the JSON specification from the first stage as its primary input. The prompt's instructions enforced a strict dichotomy between invariant and variable elements:
  - **Invariant Constraints:** The prompt explicitly mandated the preservation of the core attributes defined in the specification: the skill identifier, the learning objective, the logical solution structure, the cognitive difficulty level (A/B/C), and the pedagogical function of each distractor (e.g., “targets confusion of the sine and cosine rules”).
  - **Variation Instructions:** The prompt permitted and directed systematic changes only to surface features. It instructed the model to vary all numerical parameters within a defined range (e.g., ±30%−50%), to alter contextual elements such as names or scenarios, and to change specific geometric or algebraic forms while ensuring the underlying relational structure and required solution method remained identical.
  - **Output Formatting Rules:** The prompt enforced linguistic and presentational standards, requiring output in natural, textbook-appropriate Kazakh, the use of LaTeX for mathematical notation, and the randomization of the correct answer's position within the four-option multiple-choice format.

These prompts were iteratively refined through three cycles of testing with subject-matter experts to ensure accurate mapping to the UNT curriculum and to optimize the clarity and reliability of the LLMs' outputs. This two-prompt architecture formalized the expert-driven item design logic into a reproducible, automated workflow, ensuring that generation was both scalable and anchored to the pedagogical and psychometric requirements of the high-stakes UNT context.

### Human-AI collaborative workflow

**Figure 1:**
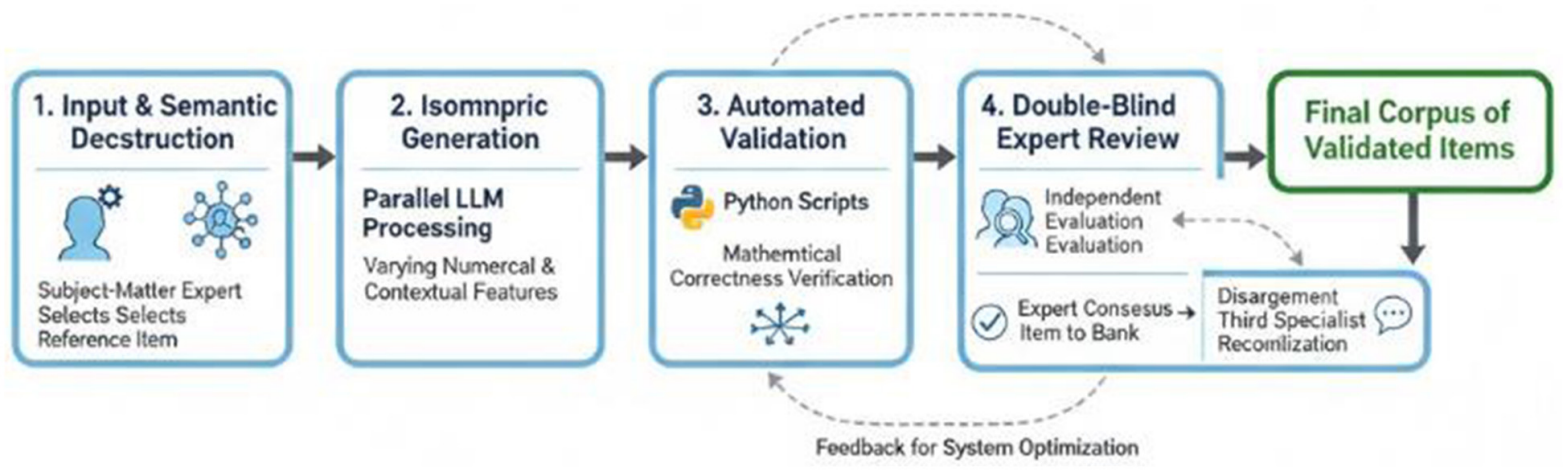
Human-in-the-loop workflow for AI-assisted item generation in Kazakhstan's Unified National Testing (UNT).

The integrated workflow for AI-assisted item generation follows a structured human-in-the-loop pipeline, illustrated in Figure 1. This iterative process ensures both the scalability of automated generation and the quality assurance of expert oversight through five sequential stages:

1. **Input & Semantic Deconstruction:** A subject-matter expert selects a validated reference item from the UNT bank. The item is semantically deconstructed by a hybrid LLM system to extract its invariant logical-mathematical core.
2. **Isomorphic Generation:** Using the structured specification, the system generates multiple isomorphic variants through parallel LLM processing, systematically varying numerical parameters (±30%−50%) and contextual elements while preserving the conceptual structure.
3. **Automated Validation:** Each generated item undergoes automated validation using Python scripts with SymPy for mathematical correctness verification.
4. **Double-Blind Expert Review:** Validated items enter independent evaluation by two subject-matter experts following standardized criteria.
5. **Reconciliation & Finalization:** Items with expert consensus proceed to the bank. Those with disagreements undergo reconciliation discussion with a third specialist.

The diagram illustrates the iterative collaboration between AI systems (for semantic analysis, generation, and validation) and human experts (for selection, review, and quality control). Solid arrows indicate the primary workflow; dashed arrows represent feedback mechanisms for system optimization.

### Expert evaluation: design, participants, and trustworthiness

This phase employed a **convergent mixed-methods design**, integrating quantitative expert validation with qualitative interviews.

### Expert panel: recruitment and composition

A purposive sample of six **(*n* =** **6) subject-matter experts** was recruited. Selection criteria were stringent to ensure high competence:

1. **Formal Qualification:** Possession of the **highest professional qualification category** for teachers in Kazakhstan.
2. **Relevant Experience:** Minimum of 8 **years** of experience teaching mathematics in **Kazakh-language** secondary schools or universities.
3. **Assessment Expertise:** Direct, hands-on experience in **developing or reviewing** mathematics items for the official UNT, ensuring familiarity with national curriculum standards and psychometric requirements.

### Quantitative validation procedure and inter-rater reliability

The 200 generated items underwent an **independent double-blind expert review**.

- **Procedure:** Each item was randomly assigned to two experts. They evaluated items using a standardized digital checklist with criteria for content accuracy, cognitive level, clarity of wording, distractor quality, and overall suitability for the UNT.
- **Inter-Rater Agreement:** To measure consistency between raters, Cohen's Kappa (κ) statistic was calculated based on their initial binary verdicts (“Accept”/“Reject”). The obtained κ = 0.78 indicates substantial agreement beyond chance (Leighton and Gierl, 2007), confirming the reliability of the expert judgments.
- **Conflict Resolution:** In cases of disagreement, a reconciliation discussion was facilitated by a third, senior methodology specialist from the National Testing Center (NTC) to reach a final consensus.

### Qualitative interview procedure and trustworthiness

Following the quantitative review, **semi-structured interviews** were conducted in Kazakh with all six experts to gain deeper insights.

### Ensuring trustworthiness (credibility, dependability, and confirmability)

We adhered to established criteria for qualitative research rigor (Nowell et al., 2017), including:

1. **Data Triangulation:** Findings from interviews were systematically cross-verified with two other independent data streams: (a) automated validation logs, and (b) quantitative expert evaluation scores. This convergence strengthened the validity of conclusions.
2. **Iterative Thematic Analysis with Team Verification:** Interview transcripts were analyzed using **inductive thematic analysis** (Braun and Clarke, 2006). Coding and theme development were performed by two researchers independently, followed by iterative discussion and consensus-building with the entire research team to audit and confirm the analytic process.
3. **Thick Description and Reflexivity:** Verbatim expert quotes are provided in the Results. The researchers' affiliation with the NTC is transparently declared, and the study's context is described in detail to allow for transferability judgments.
4. **Audit Trail:** A detailed record was maintained, documenting all steps from interview guides and audio recordings to transcripts, translation notes, codebooks, and thematic maps.

### Data analysis

A **mixed-methods analysis** framework was applied:

- **Quantitative Analysis:** Descriptive statistics (frequencies, percentages) summarized expert validation outcomes. Cohen's Kappa assessed inter-rater reliability.
- **Qualitative Analysis:** Inductive thematic analysis, as detailed above, was used to analyze interview data.
- **Integration:** Quantitative and qualitative findings were integrated during the interpretation phase to provide a comprehensive, multi-faceted understanding of the AIG system's performance.

### Ethical considerations

The study protocol received ethical approval. All expert participants provided **written informed consent**. Data were **anonymized**, and audio recordings/transcripts were stored on **password-protected, encrypted servers**. No compensation was provided for participation.

## Results

The empirical data of the study demonstrate the effectiveness and key limitations of using generative language models for the automated development of mathematics test items in the Kazakh language.

### Results of expert validation of generated items

The generated set of 200 items underwent independent content and methodological expert review. The validation results are presented in Table 1.

**Table 1:** Results of expert validation of generated items (*N* = 200).

Expert judgment	Items quantity	Share (%)
Rejected (due to substantial content/methodological flaws)	5	2.5
Recommended for use	195	97.5
*Including:*		
– Accepted without corrections	101	50.5
– Accepted after corrections	94	47.0

^*^An item was considered accepted if both independent experts issued a verdict of “Accept.” Cohen's Kappa for initial inter-rater agreement was 0.78 (substantial agreement).

The results indicate a high level of suitability of the AIG-generated content, with 97.5% of items recommended for use. However, 47.0% of the items required expert revision, underscoring the necessity of a human-in-the-loop model.

A detailed analysis of the required revisions (Table 2) showed that the primary problem areas were difficulty calibration (19.9%), item wording (17.5%), and factual content (11.6%).

**Table 2:** Distribution of items by quality category.

Quality category	Share of items (%)	Brief explanation
Fully compliant (no revision needed)	50.5	Correct in content, structure, cognitive level, and wording.
Require difficulty calibration	19.9	Difficulty level does not match target UNT requirements.
Require wording revision	17.5	Wording is unclear, ambiguous, or deviates from UNT standards.
Require clarification of content/factual accuracy	11.6	Needs context, fact, terminology, or curriculum alignment fixes.
Duplication/repeated items	0.5	Identical or nearly identical items in the generated sample.

### Examples of original and generated items

As evident from the examples presented, alterations to numerical values and contextual elements in the item stem affect only its surface formulation, while the key characteristics remain unchanged. Across all generated variants, the type of skill being assessed, the logical structure of the solution, and the answer format are preserved. Consequently, the items require examinees to perform the same cognitive operations and solve the same mathematical model, despite differences in surface parameters. Thus, the isomorphism of the automatically generated items relative to the original prototypes is confirmed.

## Discussion

The principal finding of this research is the demonstration of the dual nature of AIG technology as both a powerful accelerator and a tool requiring stringent human oversight. Quantitatively, the expert validation results are unequivocal: **97.5%** of the AI-generated items were deemed suitable for use post-review, confirming the system's capacity to produce semantically coherent “first drafts” (Table 1). This directly addresses the core operational challenge identified at the National Testing Center (NTC)—the stark disparity between a fully digital test administration platform and a predominantly manual, resource-intensive content creation process. The empirical observation of a **4–5 fold reduction** in development time (from 25–30 to 5–7 min per item) underscores AIG's significant potential to enhance productivity and scale the item development pipeline, a critical need given the UNT's requirement for frequent item bank renewal across five annual test administrations.

However, the equally salient finding is that **47.0%** of these AI-generated drafts required expert revision. This substantial proportion mandates a “human-in-the-loop” (HITL) model not as a discretionary option but as a fundamental requirement for quality assurance. As detailed in Table 2, the primary revision areas were difficulty calibration (19.9%), item wording (17.5%), and factual/content accuracy (11.6%). This pattern of limitations is not unique but aligns with international scholarship, which notes persistent challenges in AIG for calibrating cognitive complexity (Circi et al., 2023) and ensuring pedagogical and cultural validity in high-stakes contexts (Yan et al., 2024).

### Qualitative insights and mechanistic explanations

The semi-structured interviews with subject-matter experts provided critical depth to these quantitative results, elucidating the *mechanisms* behind the observed limitations and strengths.

**The Pedagogical Gap in Distractor Engineering**. Experts consistently highlighted that while the AI could generate mathematically plausible incorrect options, it failed to embed the pedagogical logic essential for diagnostic assessment. As one expert noted, “*A distractor should reflect a typical student error, not just a random arithmetic mistake. The AI lacks the classroom experience to know which misconceptions are most common for a given concept.”* This explains the “factual/content” revisions in Table 2 and underscores that effective distractor design requires cognitive modeling of learner psychology, not just mathematical variation.

**Linguodidactic Fidelity in a Low-Resource Language**. For the Kazakh language, the challenge extended beyond translation to *linguodidactic adaptation*. Experts reported that LLM outputs often contained “calqued syntactic structures” from Russian or English and used terminology inconsistent with modern Kazakh textbooks. This issue directly corresponds to the 17.5% of items needing wording revisions. It highlights a broader challenge for multilingual assessment systems: AIG must be adapted to the specific morphological, syntactic, and discourse norms of the target language, particularly for agglutinative languages like Kazakh where general-purpose models may underperform.

**The Isomorphism–Innovation Trade-off and Cognitive Ceiling**. The template-based isomorphic approach proved robust for generating items at the “knowledge” and “application” levels, successfully preserving the logical-mathematical core while varying surface parameters (as illustrated in Table 3). However, experts confirmed a “cognitive ceiling”: the system struggled with items requiring “analysis” or “synthesis.” As one expert explained, “*Higher-order items require multi-step reasoning where the path isn't linear. The AI tends to increase numerical complexity rather than construct novel conceptual relationships.”* This finding corroborates the literature on the difficulty of generating items assessing complex problem-solving (Gierl and Bulut, 2021).

**Table 3:** Examples of isomorphic cloning from prototype to AI-generated variant.

No.	Original item	AI-generated isomorphic clone	Invariant core
1	**Find the sum of the roots of the equation:** 1x−2–1x−1=16*x*−21–*x*−11 = 61 **A)** −3 **B)** −1 **C)** 2 **D)** 3	**Find the sum of the roots of the equation:** 1x−5–1x−2=13*x*−51–*x*−21 = 31 **A)** −7 **B)** 3 **C)** 1 **D)** 7	**Section:** Equations **Objective:** Solve fractional-rational equations. **Difficulty Level:** Medium
2	**Find the value of the expression:** |−10|+7|−10|+7 **A)** 17 **B)** 3 **C)** −3 **D)** −17	**Find the value of the expression:** |−15|−8|−15|−8 **A)** −23 **B)** −7 **C)** 7 **D)** 23	**Section:** Numbers **Objective:** Know the definition of absolute value and compute it. **Difficulty Level:** Easy

^*^The test items were translated from Kazakh.

**Expert-in-the-Loop as a Value Multiplier, Not a Bottleneck**. Crucially, the interviews revealed that experts perceived the HITL model not as a remedial step but as a value-adding collaboration. The consensus was that AIG excelled at the “heavy lifting” of generating structurally sound variants, thereby freeing experts to focus on higher-value tasks: refining pedagogical intent, ensuring cultural relevance, and enhancing cognitive demand. “*It shifts my role from author to editor and designer,”* one expert remarked, “*allowing me to focus on creativity and quality rather than repetitive drafting.”*

### Synthesis and practical implications

These findings collectively advocate for a **hybrid augmentation model**, where AIG is positioned as a co-pilot rather than an autopilot. The technology automates the computationally intensive, routine task of isomorphic variant generation (demonstrated in Table 3), while human experts provide the indispensable pedagogical judgment, linguistic finesse, and psychometric calibration.

For the NTC and similar assessment bodies, this study yields several actionable recommendations:

1. **Phased, Domain-Specific Implementation:** Initial deployment should target subjects and cognitive levels where AIG demonstrates high reliability (e.g., mathematics at the “application” level), establishing trust and refining processes before expanding to more complex domains.
2. **Investment in Prompt Engineering and Localized Training:** The quality of output is directly contingent on input specificity. Developing and curating a library of high-quality, Kazakh-language prompt templates that encode curriculum standards and cultural contexts is essential.
3. **Upskilling of Assessment Specialists:** Successful integration requires training item developers in new competencies, including prompt engineering, rapid validation of AI outputs, and the effective integration of AI drafts into the existing test development cycle.
4. **Development of Robust Validation Protocols:** The double-blind expert review with reconciliation, as employed in this study, should be institutionalized. This must be complemented by advanced automated validation checks (e.g., for mathematical equivalence and style consistency).

## Limitations and avenues for future research

This study has several limitations that define productive paths for future inquiry. First, its scope was confined to closed-format mathematics items in Kazakh. Future work must investigate AIG's efficacy for open-ended responses, humanities subjects (e.g., history, literature), and other languages of instruction in Kazakhstan (Russian, English). Second, the study did not include a dedicated linguistic review panel, potentially overlooking subtle nuances in language quality. Third, while the study measured inter-rater agreement, a full psychometric analysis of the generated items' difficulty and discrimination in live testing remains for future work (Pugh et al., 2020).

Promising research directions include: (1) fine-tuning LLMs on high-quality, domain-specific Kazakh corpora to improve linguodidactic output; (2) exploring “cognitive model-powered” AIG to better generate higher-order items and pedagogically valid distractors; and (3) conducting comparative studies across Central Asian multilingual assessment systems to develop regionally informed best practices for AI integration.

## Conclusion

In conclusion, this study provides evidence that AIG based on contemporary LLMs is a potent tool for scaling the development of assessment content in a linguistically specific, high-stakes context. It can dramatically improve efficiency and support the sustainability of large-scale digital assessment systems like the UNT. However, its value is contingent on a structured, expert-led framework that mitigates its current limitations in pedagogical reasoning, linguistic adaptation, and cognitive calibration. The optimal path forward is not the replacement of human expertise but its strategic augmentation, forging a collaborative partnership where AI handles scale and speed, and humans ensure quality, fairness, and validity. This hybrid model offers a pragmatic and responsible blueprint for the future of AI-assisted assessment in Kazakhstan and analogous educational ecosystems worldwide.

## Data Availability

The original contributions presented in the study are included in the article/supplementary material, further inquiries can be directed to the corresponding author.
